# Online Data Collection to Evaluate a Theoretical Cognitive Model of Tinnitus

**DOI:** 10.1044/2016_AJA-16-0007

**Published:** 2016-10-01

**Authors:** Lucy Handscomb, Deborah A. Hall, Gillian W. Shorter, Derek J. Hoare

**Affiliations:** a National Institute of Health Research Nottingham Hearing Biomedical Research Unit, Otology and Hearing Group, Division of Clinical Neuroscience, Nottingham, United Kingdom; b UCL Ear Institute, London, United Kingdom; c National Institute for Mental Health Research, Australian National University, Canberra, Australian Capital Territory, Australia; d School of Nursing and Midwifery, Trinity College, University of Dublin, Ireland

## Abstract

**Purpose:**

The purpose of this article is to describe data collection considerations, methods, and response rates for a survey available both online and on paper. Methodological issues in the design of online data collection, and advantages and disadvantages of different data collection methods are discussed.

**Method:**

A survey was compiled that included 9 full or partial clinical questionnaires designed to measure different components relevant to tinnitus distress. It was completed once by 342 members of the public with tinnitus. Respondents could choose whether to complete the survey online or on paper.

**Results:**

Ninety-five percent of participants chose to complete the survey online. The advantages of an online self-administered questionnaire include low numbers of unanswered questions, convenience (particularly in a longer survey such as this), a fast return rate, and reduced expense. Age emerged as an important variable, with those opting to complete the paper-based version of the survey being older.

**Conclusions:**

Online data collection has several advantages to both participants and researchers. However, cross-sectional studies such as that presented here should also offer paper questionnaires to avoid excluding certain subgroups of the population. Ethics and reporting guidelines for Internet-delivered questionnaire studies are available. These can usefully inform study design and guide high-quality reporting.

**C**ollecting study data online via the Internet has many advantages over paper-based data collection, including cost efficiency and efficient dataset generation (Gosling, Vazire, Srivastava, & John, 2004). Online survey software makes it easy to download data directly into computer packages and greatly reduces the potential for data entry mistakes (Alessi & Martin, 2010). Survey respondents are less likely to submit incomplete questionnaires when submitted online rather than when they are required to send paper-based responses via the mail (De Rada & Domínguez, 2015). However, online data collections may also have a number of disadvantages. Gosling et al. (2004) evaluated and discussed evidence for six assumptions about online data collection studies, namely that (a) they reduce diversity of samples (mostly White men); (b) they include a preponderance of socially isolated, depressed respondents; (c) presentation format affects the results; (d) they increase nonsense responses; (e) anonymity can affect results; (e.g., multiple completions by the same participant); and (f) they lack consistency with paper-based methods. Awareness of these issues allows a researcher to take steps to combat potential threats to validity (e.g., advertising to traditionally underrepresented communities). Of these six assumptions, there is evidence to suggest that participant anonymity compromises data quality (e.g., Skitka & Sargis, 2005). However, there is mixed or little evidence to support the other five assumptions. For example, there is mixed evidence that questionnaire data collected using different methods are consistent. Muehlhausen et al. (2015) reported that patient-reported outcomes data collected online were distributed similarly to outcome data collected using paper questionnaires. However, Thorén, Andersson, and Lunner (2012), who compared findings on four clinical questionnaires completed on paper and online, found that one questionnaire, the Hearing Handicap Inventory for the Elderly (HHIE; Ventry & Weinstein, 1982), gave higher scores when completed online. This may have been an order effect; in the online version of the study the HHIE had to be completed first, whereas for the paper version, participants completed the questionnaires in whatever order they preferred. Therefore, the authors recommended that delivery format be kept stable throughout longitudinal studies (Thorén et al., 2012). In a more recent study by Weigold, Weigold, and Russell (2013), questionnaire format was associated with the amount of missing data, and the provision of additional participant information (e.g., participant address and estimated time taken to complete the questionnaire); mean questionnaire scores were not affected by format.

McKenna, Handscomb, Hoare, and Hall (2014) recently described a new cognitive behavioral model of tinnitus distress and have since employed online and paper questionnaires in a cross-sectional survey to test associations in the model. These associations will be explored using structural equation modeling and reported in future articles. In this Research Forum article, we describe data collection considerations, methods, and response rates for this survey available both online and on paper. Methodological issues in the design of online data collection, and advantages and disadvantages of different data collection methods, are discussed.

## Method

Ethical approval of this study was granted by the University of Nottingham Faculty of Medicine and Health Sciences Ethics Committee. Reporting was guided by the Checklist for Reporting Results of Internet E-Surveys (CHERRIES; Eysenbach, 2004). CHERRIES was developed to provide recommendations that authors can follow to ensure complete descriptions of e-survey methodology, and to provide the necessary detail to judge how truly representative a survey sample is.

This was a powered nonexperimental, cross-sectional closed survey study of adults with tinnitus in which data were used to test relationships between different aspects of tinnitus using structural equation modeling. The survey included questionnaires, or selected subscales from questionnaires, measuring eight domains: tinnitus-related thoughts, control beliefs, fears about tinnitus, avoidance behavior, emotional distress, attention to tinnitus, perceived magnitude of tinnitus, and overall tinnitus distress. Questionnaires were selected by the study team according to evidence that they captured a measure of the construct of interest. There were 150 questions in all. The online survey was created using Bristol Online Surveys, a web-based survey tool (http://www.survey.bris.ac.uk/). For this version, participants were required to complete the 150 questions over 15 web pages in a fixed order. As far as was practical, the format of response options and layout were equivalent for online and paper versions. All questions had to be completed before participants could advance to the next page, but they were free to revisit and change earlier responses to any questions up to the point of submission. Participants also had the option to add any general comments in a text box at the end of the questionnaire. A paper version of the survey was also produced for participants who preferred this method or who did not have access to the Internet; in this method, participants were free to complete the questions in their preferred order. The online survey was piloted using different Internet browsers to ensure that it functioned as intended and that scores were recorded appropriately.

The study was advertised online, on the British Tinnitus Association (BTA) website and social media pages, by email to members of the National Institute for Health Research, Nottingham Hearing Biomedical Research Unit (BRU) volunteer database, and in print in the BTA magazine and the BRU newsletter. Advertisements stated that the survey could be completed online or on paper. Depending on their preference, potential participants who expressed an interest in the study were sent either study information by email or by mail. The email contained the participant information sheet in PDF format and a link to the online consent form. Interested participants completed the online consent form and were thereafter sent a unique log-in for the survey. By mail, participants were sent a paper patient information sheet and consent form with a return-addressed, stamped envelope. On return of the signed consent form, the questionnaire was sent to the participants. By both methods, there was some degree of control for multiple entries.

The participant information sheet indicated that the survey would take between 60 and 90 min to complete, detailed the procedure for consent and withdrawal of consent, and provided contact details for the research team and an independent patient advice and liaison service should they have unanswered questions about this research. As an incentive, participants were invited to opt into a prize draw in which they could win a £100 retail voucher. People who made contact by email were assumed to prefer an online survey if they did not state a preference.

In order to recruit a sample of participants who were representative of a general population in terms of tinnitus severity (on the basis of Zeman, Koller, Schecklmann, Langguth, Landgrebe, & TRI Database Study Group, 2012), participants were asked to rate “How much of a problem is your tinnitus?” on a 5-point Likert scale that ranged from *not a problem* to *a very big problem*. Recruitment was then stratified according to their responses to this single question, such that different degrees of severity were proportionally represented. This strategy was considered important, given the nature of the study; there was no obligation to complete the survey, as would be the case in which participants hope to qualify for a clinical trial, for example, and there was little pressure on participants from the study team to complete the survey. If recruitment had not been stratified then the sample could have been biased toward those who were more motivated by research (i.e. those with more severe tinnitus).

Following return of their consent forms and responses to the initial question, participants were emailed a unique user log-in to access the full survey or were sent a paper copy of the survey in the mail. Each log-in could only be used once. Internet protocol (IP) addresses were not captured. When completing the online survey, a response to each item was required in order to progress to the next item. The survey could be submitted only after all 150 questions were answered. ID numbers of people who logged on but did not complete the survey were listed by the software but their responses were not retained. Time taken to complete the questionnaire was not captured. Electronic data were stored on a secure university server and backed up daily. Paper data were stored in a locked cabinet in a locked office on university premises.

For guidance on ethical considerations related to Internet-mediated research, including confidentiality and security of online data, procedures for obtaining valid consent, and procedures for ensuring withdrawal rights and debriefing, the reader is directed to the British Psychological Society (2013).

## Results and Discussion

The survey was advertised May–December 2014. During that time, 536 people expressed an interest in participating. Of those, 40% stated they had heard about the study via an email sent to participants registered on our departmental database, and 26% stated they had heard about it via the BTA website, social media pages, or in its magazine. Three percent of participants heard about the study at their local tinnitus self-help support group. The remaining participants did not specify how they became aware of the study.

### Study Population

From the 536 expressions of interest, 436 participants (81%) consented to participate, and 342 (78%) completed the survey. Of those completing, 323 (95%) chose the online format and 19 (5%) chose the paper format. The proportion who consented to participate but did not go on to complete the full survey was 21% in both cases (87/410 in the online group and 5/24 in the paper group). Of the online group, 11% began the survey but did not submit it and 10% did not begin it. The median number of days between being sent a link to the survey and completing it online was 1 day (range = 0–70 days). Most participants (54%) submitted their survey on the same day or the day after they were sent the link to it. A single reminder was sent to 44 participants who consented but did not participate within four weeks, and 23 of those participants subsequently completed the survey. After giving online consent, two participants emailed the lead researcher to withdraw that consent; one reread the information sheet and realized he was not eligible because of his age, and the other felt she did not like answering questions about her tinnitus.

There were no proportional differences in sex by survey format (online: 173 men, 150 women; paper: 13 men, 6 women). The mean age of online respondents was 54.1 years (range = 21–83), and the mean age of paper respondents was 67.5 years (range = 43–87). This difference was statistically significant, *t*(340) = *−*4.308, *p* < .001, suggesting the online survey reached or was preferred over the paper questionnaire by the younger adult population.

Mean scores on questionnaires used in the survey, completed either online or on paper, are given in Table 1. A one-way between-groups multivariate analysis of variance was performed to identify any differences in mean scores on the questionnaires that made up the survey. There was no statistically significant difference between online and postal groups on the combined questionnaires, *F*(8, 330) = 1.072, *p* = .383; Wilks’s lambda = .975. Thus, despite the online group being younger, overall tinnitus or general distress by any measure was comparable between the groups.

### Practical Benefits of Using an Online Survey

The online survey was designed such that participants could not proceed to the next page until a response to every item had been entered. One advantage for data collection was that this completely avoided missing data. In contrast, there was a small amount (0.2%) of missing data among the mailed responses. One participant did not respond to four items, one participant did not respond to two items, and two participants did not complete a large number of items; all were contacted by the lead researcher asking them to fill in the missing items. Studies that involve larger numbers of people completing paper questionnaires typically report larger amounts of missing data; for example, Parker and Dewey (2000) reported that 38% of the 343 questionnaires returned in their occupational therapy paper survey had missing responses. That said, several online respondents commented that they would have liked to omit some questions because they did not consider them applicable. By obligating people to answer all questions there is perhaps a risk that some meaningless or inaccurate responses are included in the data, or that, as was the case for at least one of our participants, the relevance or nature of questions they are being “forced” to respond to leads them to abandon the questionnaire altogether. When designing survey-based studies, there is, therefore, a necessary trade-off to consider between the need for completeness of the dataset, as is required for some modeling approaches, and the need for accuracy.

Online data had no errors in data entry; scores could be exported as .csv files and read in Excel. However, caution is still advised. Conversion of text to numerical responses, and reverse scoring of certain questions, was required after export, introducing potential for human error. An audit of the paper-based data transcribed into Excel revealed a small number of errors, most of which related to reverse scoring individual questions.

Internet-delivered surveys also bring substantial cost savings. As an example, if the 972 emails sent (including consent forms and links to access the online survey) had been posted out as letters, the associated stationery and postage costs would have been about £2,000.

### Practical Benefits of Using a Postal Survey

Participants who completed the postal survey commented that this option was important to them. Five participants in the Scottish Highlands reported having an unreliable Internet connection and completed the survey on paper and posted their responses. Some older participants indicated they felt uncomfortable with the Internet. Although 80% of the general population of the United Kingdom use the Internet (Dutton & Blank, 2013), only 61% of those age 50–62, and 30% of those age 63–74, use it (Henshaw, Clark, Kang, & Ferguson, 2012). It is likely that those over 74 years of age are even less likely to use the Internet. Population is therefore an important consideration when devising questionnaire studies.

## Conclusion

The Internet is a useful tool for large-scale data collection. In our study, it reduced the monetary and human resources required, and reduced human error that may result from transcription of paper-based data into a database. There is some evidence that the format of collection (paper vs. online) affects clinical questionnaire data but here we did not find a difference in questionnaire scores between online and paper groups. Although Internet use is widespread, and there is a clear preference by most people to complete large surveys in an online format rather than on paper, providing the option to complete a paper-based questionnaire is important and, indeed, essential for results to include the 20% of participants who are not online and to be fully generalizable. Designing online surveys with due consideration of existing ethics guidance of Internet-mediated research and reporting according to the CHERRIES guidelines, is recommended.

## Figures and Tables

**Table 1:** Mean questionnaire scores for online and paper responses.

Concept measured	Questionnaire	Mean score (*SD*): Online responses	Mean score (*SD*): Paper responses	Maximum possible score
Thoughts	Tinnitus Cognitions Questionnaire (Wilson & Henry, 1997)	43.98 (17.04)	41.72 (20.34)	104
Emotional distress	Clinical Outcomes in Routine Evaluation (Evans et al., 2000)	33.10 (22.69)	30.40 (14.77)	136
Attention and monitoring	Tinnitus Vigilance & Awareness Questionnaire (Cima et al., 2011)	39.25 (19.63)	44.80 (21.89)	90
Control beliefs	Illness Perception Questionnaire (Weinman et al., 1996)	19.83 (8.74)	20.77 (9.22)	60
Tinnitus-related beliefs	Fear of Tinnitus Questionnaire (Cima et al., 2011)	9.49 (6.80)	10.16 (5.74)	40
Safety behavior	Tinnitus Fear Avoidance Scale (Kleinstäuber et al., 2013)	15.86 (11.60)	17.90 (13.76)	66
Tinnitus magnitude	Tinnitus Magnitude Index (Schmidt et al., 2014)	17.92 (7.99)	17.95 (8.08)	30
Tinnitus distress	Tinnitus Function Index (emotional subscale) (Meikle et al., 2012)	8.47 (8.85)	10.48 (9.28)	30
Tinnitus distress	Tinnitus Reaction Questionnaire (Wilson et al., 1991)	26.44 (25.06)	29.95 (25.99)	104
